# The Advanced Practitioner and Collaborative Practice in Oncology

**Published:** 2015-11-01

**Authors:** Sandra E. Kurtin, Mary Peterson, Paige Goforth, Megan Brafford May, Pamela Hallquist Viale, Wendy J. Smith, Deborah Rust, Carolyn Grande, Nancy M. Nix, Catherine S. Bishop

**Affiliations:** 1University of Arizona Cancer Center; 2St. David’s South Austin Medical Center; 3Wellmont Cancer Institute; 4Baptist Health Lexington; 5University of California, San Francisco; 6Meniscus Educational Institute; 7Genentech GI & Lung Franchise; 8Abramson Cancer Center, Hospital of the University of Pennsylvania; 9St. Joseph’s/Candler Health System; 10Johns Hopkins Kimmel Cancer Center at Sibley Hospital

The term "advanced practitioner" (AP) refers to health-care professionals who have completed advanced training in nursing or pharmacy or who have completed training as a physician assistant (PA). Educational requirements, training, the scope of practice, governing boards (state/national), national certification requirements and organizations, and collaborative practice agreements vary by role (Table 1).

**Table 1:**
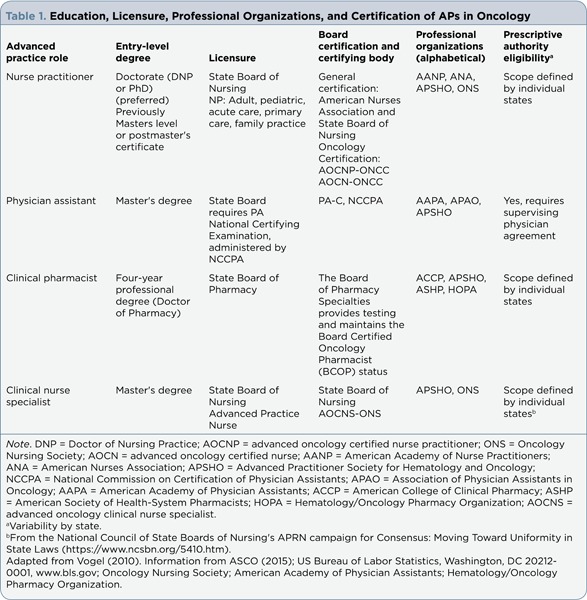
Education, Licensure, Professional Organizations, and Certification of APs in Oncology

Oncology APs are licensed health-care providers with expert knowledge and advanced skills in managing patients with hematologic or oncologic diagnoses across the health and illness continuum in a variety of health-care settings. Nurse practitioners (NPs), physician assistants, clinical pharmacists, clinical nurse specialists, and nurses with advanced degrees comprise the core of APs in oncology. Although the duties performed vary according to practice setting and collaborative agreements, APs in oncology manage patients requiring complex procedures and treatments.

The AP workforce dedicated to oncology comprises a mix of roles across practice settings and states. This specialty group of APs provide an array of critical services for cancer care (Table 2). Importantly, the majority of APs have prescriptive authority, which is necessary for cancer treatment and prevention and management of adverse events. The varied scope of practice is largely explained by individual state practice acts with some guidance from national AP organizations. The individual scope-of-practice documents are available from the different professional organizations for each AP role (Table 1). Although not all APs participate fully in each role, the focus is on a collaborative practice model. Collaborative practice implies involvement from all members of the interdisciplinary team and aims to achieve the best outcome for each patient based on practice guidelines and individualized patient and caregiver assessment.

**Table 2:**
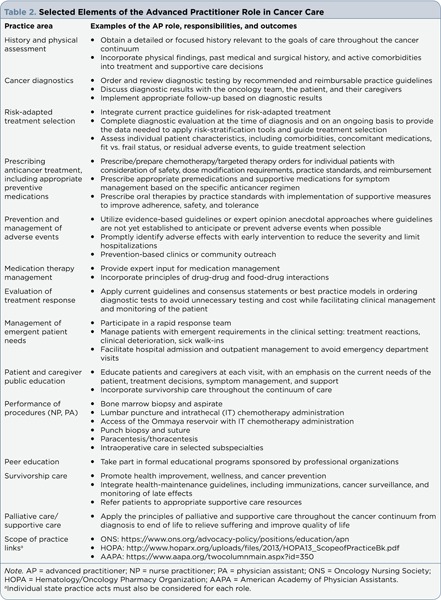
Selected Elements of the Advanced Practitioner Role in Cancer Care

The American Society of Clinical Oncology (ASCO) Annual Report calculates that there are approximately 3,000 APs working in oncology today. Although not all APs in oncology belong to a professional association, many do. Oncology certification is currently available for selected AP roles. Oncology certification for Advanced Practitioners in Oncology with nursing degrees is provided by the Oncology Nursing Certification Corporation, a subsidiary of the Oncology Nursing Society (ONS). Board Certified Oncology Pharmacist (BCOP) status is obtained from the Board of Pharmacy Specialties (BPS). Certification in oncology practice for PAs is not currently available.

The exact number of APs in oncology is uncertain. The ONS estimates that in 2015, their membership includes 2,601 NPs and 1,173 clinical nurse specialists (CNSs). The number of pharmacists specializing in oncology practice is estimated to be nearly 2,400 based on Hematology/Oncology Pharmacy Organization (HOPA) membership. The ASCO annual practice census noted 2,752 NPs and 1,136 PAs, with the majority working in academic settings, although an increase in the number of APs in physician-owned and hospital-based practices is expected to increase based on this survey (ASCO, 2015). The need for research to more accurately identify the number of APs working in oncology in varied settings and roles is essential to understand the implications for oncology practice and the health-care challenges of the future.

## SCOPE OF THE PROBLEM: THE CHANGING CANCER CARE ENVIRONMENT

**Figure 1:**
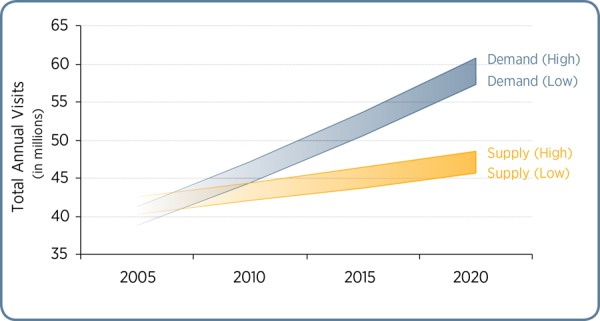
Projected supply (visit capacity) and demand for visits, 2005-2020 AAMC Center for Workforce Studies. (2007, March). Forecasting the supply of and demand for oncologists.

The 2015 ASCO report places an emphasis on practice trends, workforce composition, health systems innovation, regulatory compliance, and the financial realities of cancer care today (ASCO, 2015). Among the most pressing issues highlighted in the report was a growing cancer population, increased complexity of the care provided, and an oncology workforce that is projected to fall short of the expected demand (Figure 1). In a recent survey of 22,000 oncologists, 11,700 medical oncologists were estimated to provide direct care, managing the majority of cancer patients over extended periods of time (ASCO, 2015). Some key contributing factors to this predicted shortfall of providers and increasing complexity of cancer care delivery include:

(1) Implementation of the Affordable Care Act (ACA) with an increasing number of individuals gaining access to insurance through an expansion of insurance options;

(2) Baby boomers expanding the older adult population, with Medicare as the primary insurance plan;

(3) A growing number of cancer survivors due to improvement in cancer detection, risk-adapted treatment strategies, supportive care, and palliative care (American Cancer Society, 2014);

(4) The increasing cost of care requiring a shift in practice models and integration of formalized programs for preauthorization and reimbursement;

(5) An aging hematologist/oncologist workforce (50% over the age of 50), with a shift toward group practices in urban settings (> 90%; ASCO, 2015). A decrease in oncology coverage in rural settings together with continued low enrollment of ethnic minorities in hematology/oncology fellowship programs contribute to health disparities in cancer care;

(6) Cancer care initiatives set as standards of care or required for certification necessary to achieve designation or improve revenue;

(7) Meaningful Use as a part of the ACA-mandating benchmarks for the use of the electronic health record (EHR) and patient-reported outcomes (PROs);

(8) Commission on Cancer (COC): The American College of Surgeons published "Cancer Program Standards 2012: Ensuring Patient-Centered Care" (2012), establishing new requirements around patient-centered needs and expanding the focus on improving the quality of care and patient outcomes. More recently, the COC has set a standard for distress screening for every cancer patient and their caregivers across the continuum of care (Lazenby, Dixon, Bai, & McCorkle, 2014; Schilli, 2014; Zebrack et al., 2015);

(9) Survivorship Care: The Institute of Medicine (IOM), ASCO, and the COC have set guidelines for survivorship care. Cancer survivors are projected to exceed 19 million by 2024 (American Cancer Society, 2014);

(10) Palliative Care: The IOM released its report "Improving Palliative Care for Cancer" in 2000 (IOM, 2000). ASCO published a provisional clinical opinion in 2012, recommending that palliative care be integrated into the care of every patient with cancer at the time of diagnosis. The National Consensus Project put forth its Clinical Practice Guidelines for Palliative Care (2013), a set of nationally recognized guidelines. These guidelines include quality measures and the eight domains of palliative care. The National Comprehensive Cancer Network (NCCN) published the first clinical practice guidelines for palliative care in 2013 (NCCN, 2015). It is important to note that on July 8, 2015, Medicare released its proposed physician fee schedule covering the 2016 calendar year. Among notable elements of the rule is a proposal to pay for advanced care planning services. Implementation of the Medicare legislation, in conjunction with a more focused effort for program development and measurement of patient outcomes, may facilitate broader implementation of palliative and supportive care and advance care planning.

## PROPOSED SOLUTIONS

Among the solutions suggested by ASCO and other health-care organizations to address some of the current challenges in the delivery of oncology care is the integration of APs into cancer care across practice settings. Integration of APs using a collaborative practice model is proposed as an ideal solution to the challenge of complex cancer care across multiple settings with the anticipated shortfall of practicing hematologists and oncologists.

**Figure 2:**
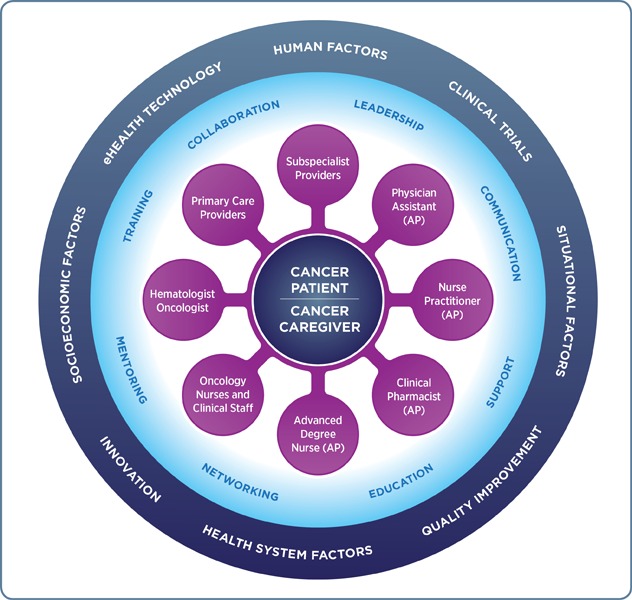
Collaborative practice in oncology is a dynamic process focused on interdisciplinary support of patients and their caregivers with a broad range of health-care providers. The AP in oncology plays a critical role in the collaborative management of patients and their caregivers. Ongoing education, training, mentorship, networking, and communication are necessary to cultivate and maintain a collaborative practice model. Integration of resources from each practice setting, community organizations, e-health technologies, and advocacy groups is essential. Human factors, health system factors, situational factors, and socioeconomic factors are ever-changing within the continuum of care and must be considered in designing tailored patient and caregiver support. Collaborative practice is endorsed by professional organizations that provide education, training, and advocacy. Ongoing clinical and practice research provides the foundation for continued adaptation to the rapidly changing trends in oncology practice. Regulatory and quality improvement measures must be integrated throughout. (Created by Sandra Kurtin)

Collaborative practice implies effective working relationships with physicians and other members of the health-care team (Figure 2). The degree of autonomy is determined not only by the scope of practice, but by expertise, knowledge, and skills demonstrated over the course of the AP’s professional practice. Thus, a degree or certification does not imply immediate independence or autonomy; this must be earned through practice, collaboration, and lifelong learning.

The complexity and sometimes frenetic pace in oncology practice today, together with some policy changes mentioned previously, has placed cancer providers at risk. As previously mentioned, there are too few oncology providers in practice, and to meet the need of the expanding population, efforts for recruitment and retention of oncology providers will be essential. Provider and patient satisfaction are imperative to promote continuity of care and staff retention.

The cost of care is a primary concern in oncology today (ASCO, 2015). Collaborative practice models that provide mechanisms for revenue generation while reducing unnecessary costs to patients through application of clinical practice guidelines will promote patient and provider satisfaction.

Schulman (2013) suggested several goals for collaborative practice in oncology: (1) Improved patient care, (2) increased clinical productivity, (3) improved access for patients, (4) urgent care patient management, (5) care of the long-term cancer patient, and (6) coverage for the academic physician. Towle and colleagues (2011) suggested similar roles for the AP in a collaborative practice model, including (1) assisting patients during treatment visits; (2) pain and symptom management; (3) follow-up care for patients in remission (survivorship care); (4) patient education and counseling; (5) end-of-life care; and (6) ordering chemotherapy. The underlying theme in these publications is that a collaborative practice model, with oncologists and APs in oncology working together to the extent of their training and licensure, can improve patient and provider satisfaction as well as safety and will serve to increase productivity and revenue (Brown, 2011; Hinkle et al., 2010).

**Figure 3:**
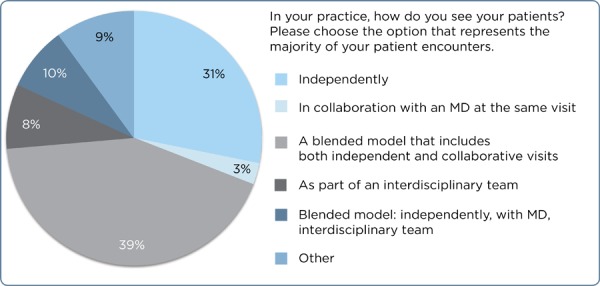
Collaborative practice models represented by the APSHO practice survey (N = 192).

Varied collaborative practice models are currently in use based on the needs of the practice, the patient volume, the skills, and the training of the physician and the AP (Figure 3). Each has implications for billing and productivity. The key to the efficient integration of the AP in oncology into a collaborative practice model is a careful assessment of skills and knowledge about oncology practice.

Although many educational programs for APs include cancer screening, prevention, diagnostic evaluation, and general cancer care in their curriculum, most APs will have limited exposure to managing care for cancer patients and the specific therapies. This creates a significant learning curve. Based on a recent survey conducted by the Advanced Practitioner Society for Hematology and Oncology (APSHO), the majority of APs in oncology do not have a formal orientation program, but rather on-the-job training with the expectation that they perform care related to knowledge that is not incorporated into their training (Kurtin, Viale, Hylton, Campen, & Vogel, 2015). Furthermore, productivity assessment models for APs are lacking (Moote, Nelson, Veltkamp, & Campbell, 2012).

Given the complexity of cancer care today, the AP in oncology will require specialty education and training, with a focused effort in the onboarding process and continuing education over the course of their career. The challenge is to fill the educational gaps for all members of the interdisciplinary oncology team. To do this while effectively integrating advances in science and practice, and maintaining excellence in cancer care amid the ever-changing policies and practice environments, will require lifelong learning and innovative approaches to education.

## THE ADVANCED PRACTITIONER SOCIETY FOR HEMATOLOGY AND ONCOLOGY

The Advanced Practitioner Society for Hematology and Oncology, or APSHO (www.apsho.org), is a newly formed society for nurse practitioners, physician assistants, pharmacists, clinical nurse specialists, and other advanced degree nurses. The society was launched in January 2014 in response to identified educational and professional development needs for the AP in oncology. At the core of the APSHO mission is facilitating collaborative practice in oncology care across the cancer care continuum and a variety of practice settings. As such, APSHO aims to improve the quality of care for patients with cancer. Membership in APSHO is inclusive, encouraging a diverse group of APs and affiliates to foster communication, education, and preparation for advances in oncology care, including collaboration with established specialty organizations that currently focus on individual AP roles. A total of 550 APs became APSHO members within the first year of the organization. A description of the APSHO mission statement and details about the committee structure are presented in Appendix A.

## THE JOURNAL OF THE ADVANCED PRACTITIONER IN ONCOLOGY

*The Journal of the Advanced Practitioner in Oncology*, or JADPRO (www.advancedpractitioner.com), is the official journal of APSHO, serving to improve the quality of care for patients with cancer, support critical issues in advanced practice in oncology, and recognize the expanding contributions of APs in oncology. Each issue of JADPRO is sent to nearly 10,000 readers. A description of the JADPRO mission statement, editorial board, and publishing team is included in Appendix B.

## JADPRO LIVE AT APSHO

JADPRO Live (www.jadprolive.com) is now the official annual meeting for APSHO. The continuing education (CE)–accredited sessions at JADPRO Live presented throughout the conference include didactic, interactive, evidence-based, and fair-balanced content targeted to APs in oncology. JADPRO Live weaves the collaborative practice model throughout the sessions provided. Advanced practitioners and physicians come together to discuss current treatment options and advances in the care of the patient with cancer, describe key practice initiatives that are essential to the AP, and identify means to improve collaboration. The ultimate goal is to improve patient outcomes and the quality of care.

A panel discussion held at the inaugural meeting in January 2014 included leadership from ASCO, the American Society of Hematology (ASH), the NCCN, and the American Society of Radiation Oncology (ASTRO). The panel, moderated by APSHO founding board member Pamela Hallquist Viale, discussed the current and future challenges faced by oncology professionals and identified strategies to address the anticipated shortfalls in the oncology workforce.

A quote by Dr. Steven Allen, representing ASH Education, emphasized the importance of collaborative practice and shared goals for education, training, and maintenance of expertise for the AP in oncology: "Our program could not function and maintain its high standards without the assistance of our advanced practice colleagues."

Dr. Robert W. Carlson, representing the NCCN, mentioned that he has worked collaboratively with APs in oncology his entire career, emphasizing their expertise in symptom management and as "protectors of patient safety." Dr. Carlson added, "You need to set the standards incredibly high and insist on excellence, being able to trust an AP in oncology to triage patients quickly and appropriately is essential."

## THE APSHO PRACTICE SURVEY

With representation from 37 of 50 states, 192 APSHO members completed a practice survey in late 2014. The majority of respondents (77%) reported more than 10 years of oncology experience, with 22% reporting more than 20 years of experience and 23.7% reporting less than 5 years of experience.

Respondents reported working more than 40 hours per week (63%), with a minority working part-time (13% working 30 hours or less; 25% working 30 to 40 hours per week). The high numbers of hours worked are likely a result of the increasing complexity of oncology care together with extended survival for patients requiring survivorship care or ongoing treatment.

The most common practice setting for APs in this survey (> 50% of the time) was outpatient oncology settings with no bone marrow transplant coverage (54%, n= 87/160). Fewer APs reported working in either combined inpatient-outpatient practices (5%, n= 7/128) or inpatient-only models (7.5%, n= 10/136). Blended practice models (39%, n= 68), independent visits (31%, n= 53), and rounding with the interdisciplinary team (18%, n= 32) were the most common practice models, which is not surprising given the highly experienced workforce represented in this study (Figure 3). Of the APs surveyed, 68% worked with one to five physicians.

The majority of respondents indicated that they billed for services either independently (27.6%), through a combined model of independent and incident to billing (30.5%), or through incident to only (17.2%). Twenty-five percent of respondents did not bill for service.

The scope of practice for the APs in this survey varied, with the majority having full prescriptive authority (63.4%, n= 109/172). State practice laws (15.1%, n= 26/172) were reported as barriers to prescriptive authority. Ordering chemotherapy is a key component of oncology practice. The majority of APs in this survey indicated that they worked collaboratively with the hematologist/oncologist in developing the chemotherapy plan (67%, n= 67/172). When considering hormonal therapy (57%, n= 98/172) or bisphosphonate treatments (57%, n= 98/172), a majority of APs were able to order these agents independently. Some had standing protocols in place in their institution (11.6%, n= 20/172 in both groups). Most APs were autonomous when performing procedures, with the most common procedures being bone marrow biopsies, Ommaya reservoir access for chemotherapy, lumbar punctures, punch biopsies, and minor suturing (Table 2).

**Figure 4:**
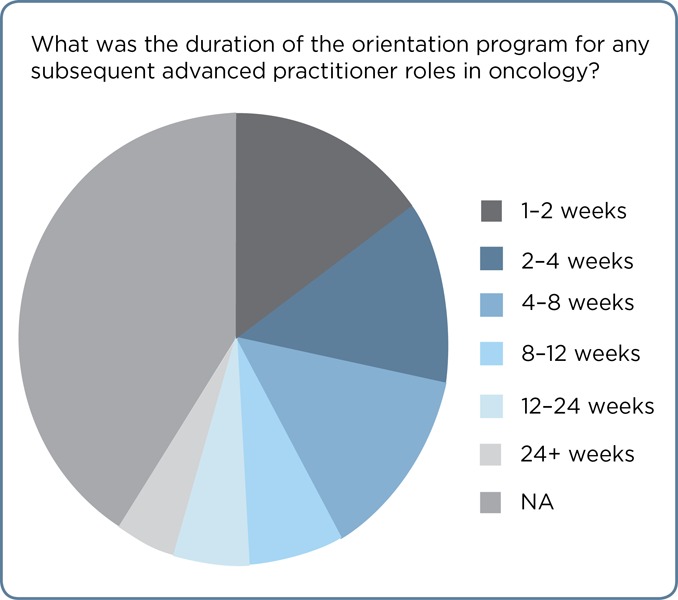
Duration of orientation program for any subsequent AP role, according to APSHO practice survey (N = 192).

Importantly, the education and training models reflected in this survey emphasize the lack of a consistent approach for entry into practice. The most common model was on-the-job training without a formal plan of orientation (55%, n= 94/171), and many respondents indicated their practice only hired APs with oncology experience (12%, n= 21/171). A minority of APs in this survey indicated that their oncology practice used a formal training program (11.7%, n= 20/171). When asked what type of training was used when they entered the oncology workforce, the majority of respondents (77%, n= 132/171) indicated that an informal on-the-job training model was used. For those still in active oncology practices, the training period has remained less than 8 weeks for new hires (72%, n= 74/103; Figure 4).

**Figure 5:**
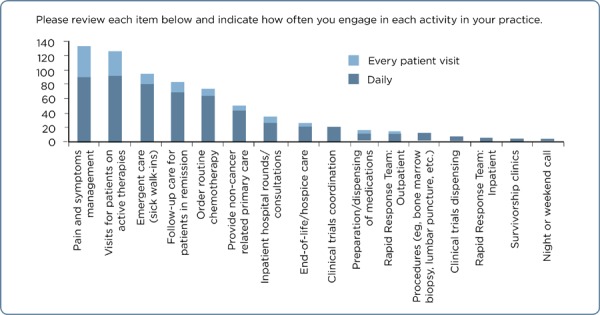
Activities engaged in at every visit or daily, according to APSHO practice survey (N = 192).

In this survey, APs were asked to rate activities by how often they engaged in each activity. Pain and symptom management visits for patient on active therapies, follow-up care for patients in remission, emergent care (sick walk-ins), ordering routine chemotherapy, providing non-cancer-related primary care, and participating in inpatient hospital rounds were identified as components of every visit or performed daily (Figure 5). Many of the APs in this survey (54%, n= 88/162) participate in tumor boards regularly and serve on various practice-based committees.

**Figure 6:**
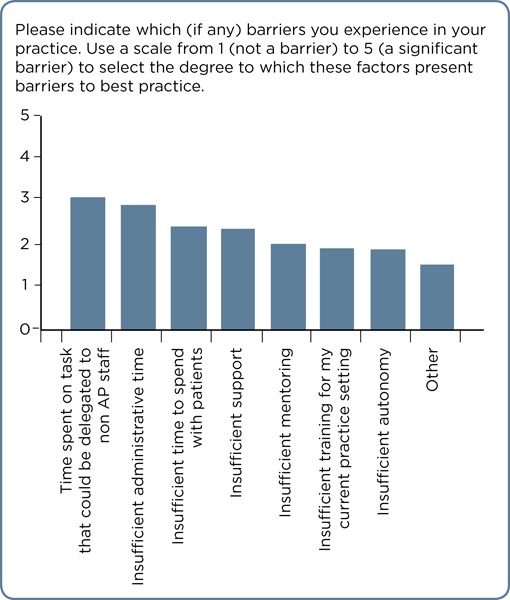
Barriers to oncology advanced practice from APSHO practice survey (N = 192).

Respondents were asked to describe barriers to their practice, rating the items on a scale of 1–5, with 5 representing a significant obstacle. Time spent on tasks that could be delegated to a non-AP staff member (ranking average 2.99, n= 163) ranked the highest, with insufficient administrative time (2.79), insufficient time to spend with patients (2.32), inadequate support (2.29), and inadequate training for my current practice (1.96) ranked among the top 5 barriers (Figure 6).

## LIMITATIONS

This online survey was sent to APSHO members and is based on self-reported data. The respondents were predominantly nurse practitioners. Not all respondents completed every question. Therefore, the number of responses to individual questions may vary, as indicated in the specific data reported. It is uncertain whether these data represent the AP in the oncology workforce as a whole or APs who are motivated to engage in APSHO as a new organization focused on the AP in oncology and collaborative practice.

## CONCLUSION

The complexity of delivering cancer care is increasing steadily. There is an anticipated workforce shortfall, namely practicing hematologists and oncologists. The number of cancer survivors is steadily increasing, with 19 million survivors anticipated by 2024. Advanced practitioners in oncology, including nurse practitioners, physician assistants, clinical pharmacists, and other nurses with advanced degrees, represent a workforce poised to fill this gap. Using a collaborative practice model, hematologists and oncologists together with APs have the opportunity to develop programs that will adequately address the complex needs of patients with cancer and their caregivers across the continuum of care. Organized programs to address the educational and training needs of the AP in oncology will be necessary. Continued collaborative efforts among professional organizations that represent cancer providers are imperative.

APSHO represents the AP within the collaborative model, providing support and education in the increasingly rewarding yet complex oncology arena.

## Appendix A: The Advanced Practitioner Society for Hematology and Oncology (APSHO)

**Mission**

Our mission is to improve the quality of care for patients with cancer by supporting critical issues in educational, clinical, and professional development for advanced practitioners in hematology and oncology.

**Collaborative Practice**

An interdisciplinary team approach to cancer treatment offers the best hope for our patients’ cure, quality of life, and survivorship.

**APSHO Committees, Publications, and Educational Initiatives**

Three initial committees have been formed to support the initiatives of APSHO: Education, Communications, and Membership.

**Figure 7:**
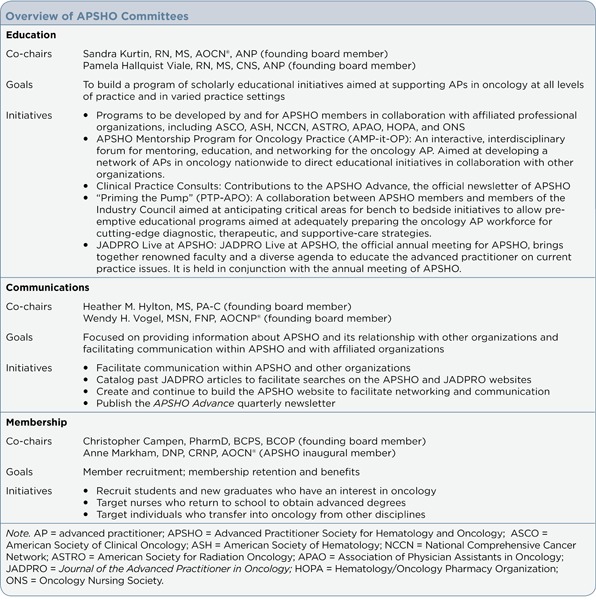
Overview of APSHO Committees

## Appendix B: *Journal of the Advanced Practitioner in Oncology* (JADPRO)

**Mission**

The mission of the *Journal of the Advanced Practitioner in Oncology* (www.advancedpractitioner.com) is to improve the quality of care for patients with cancer, support critical issues in advanced practice in oncology, and recognize the expanding contributions of advanced practitioners in oncology. JADPRO is indexed in PubMed Central.

**Objectives**

The primary objectives of JADPRO are as follows:

(1) To publish topics across the cancer trajectory for nurse practitioners, physician assistants, clinical nurse specialists, advanced degree nurses, and pharmacists

(2) To support professional development of the advanced practitioner in oncology

(3) To promote interprofessional collaboration

(4) To uphold the highest ethical and professional standards

(5) To provide information that will enhance the quality of care for patients with cancer

**JADPRO Editorial Board**

The Editorial Board includes representation for APs in varied roles across diverse practice settings.

**Editor-in-Chief:** Pamela Hallquist Viale, RN, MS, CNS, ANP

**Associate Editors**

Paula Anastasia, RN, MN, AOCN®

Cedars-Sinai Medical Center

Jeannine M. Brant, PhD, APRN, AOCN®

Billings Clinic Cancer Center - ICC

Christopher J. Campen, PharmD, BCPS, BCOP 

*Arizona Cancer Center*

University of Arizona

Beth Eaby-Sandy, MSN, CRNP, OCN®

Abramson Cancer Center

Denice Economou, RN, MN, CNS, AOCN®, CHPN

City of Hope National Medical Center

Carolyn Grande, CRNP, AOCNP®

Abramson Cancer Center

Hospital of the University of Pennsylvania

Heather M. Hylton, MS, PA-C

Memorial Sloan Kettering Cancer Center

Sandra E. Kurtin, RN, MS, AOCN®, ANP-C

Arizona Cancer Center, University of Arizona

Lydia T. Madsen, PhD, RN, AOCNS®

MD Anderson Cancer Center

Constance Visovsky, PhD, RN, ACNP-BC

University of South Florida

Wendy H. Vogel, MSN, FNP, AOCNP®

Wellmont Cancer Institute

Steven H. Wei, MS, MPH, PA-C

MD Anderson Cancer Center

Rita Wickham, PhD, RN, AOCN®

MD Northern Michigan University

**Editorial Board**

Ayman Alnems, RN, MSN, CNS

Dayne Alonso, PA-C 

Catherine S. Bishop, DNP, NP, AOCNP®

Margaret Firer Bishop, RN, MS, APRN

Tami Borneman, RN, MSN, CNS

Ingrid Bowser, MS, APRN-BC, ADM-BC, AOCNP®

Carrie F. Daly, RN, MS, APN

Mary Caroline Deigert, BS, MPAS, PA-C

Deena Damsky Dell, MSN, RN-BC, AOCN®

Hollie Devine, MSN, RN, ANP-BC, AOCNP®

Anecita Fadol, PhD, RN, FNP-BC, FAANP

Sherry Goldman, NP, RN, CBCN

Amy Goodrich, MSN, CRNP-AC

Jill M. Gore, MPAS, PA-C 

Kristina M. Gregory, RN, MSN, OCN® 

Marilyn L. Haas-Haseman, PhD, ANP-BC

Heather Hampel, MS, LG

Catherine Harvey, DrPH, RN

Jessica L. Kozuki, RN, MSN, NP, AOCNP®, CNS

Kristen Kreamer, CRNP, MSN, AOCN®, APRN, BC

Gail Kwarciany, RN, MSN, BC, OCN®, AOCNS®

Eva Lu T. Lee, MSN, RN, ANP-BC

Joanne Lester, PhD, CRNP, ANP-BC, AOCN®

Kelley D. Mayden, MSN, FNP, AOCNP®

Ali McBride, PharmD, BCPS

Sandra A. Mitchell, PhD, CRNP, AOCN® 

Susan Newton, RN, MS, AOCN®, AOCNS®

Nancy M. Nix, PharmD, BCPS, BCOP 

Patricia Palmer, RN, MS, AOCNS®

Linda Penwarden, MN, RN, AOCN®

Linda Person, RN, MSN, AOCN®

Todd Pickard, MMSc, PA-C

Julie Ponto, PhD, RN, ACNS-BC, AOCNS®

Karen Roesser, RN, MS, AOCN®

Robin Sommers, DNP, ANP-BC, AOCNP®

Mady Stovall, RN, MSN, ANP-BC

Eric D. Tetzlaff, MHS, PA-C

Carol S. Viele, RN, MS, CNS, OCN®

Julie G. Walker, MSN, RN, FNP-C

Laura Zitella, MS, RN, ACNP-BC, AOCN®

**Publishing**

JADPRO is published by Harborside Press

Executive Vice-President, Editorial: Conor Lynch

Managing Editor: Claudine Kiffer

Acquisitions Editor: Kelley Moore, RN

President: Anthony Cutrone
